# FLEXIBILITY MEETS COMPLEXITY: LESSONS FROM PRACTICE-BASED IMPLEMENTATION OF A DEMENTIA CARE MODEL

**DOI:** 10.1093/geroni/igae098.1344

**Published:** 2024-12-31

**Authors:** Halima Amjad

**Affiliations:** Johns Hopkins University School of Medicine, Baltimore, Maryland, United States

## Abstract

People living with dementia (PLWD) are among the highest-need and highest-cost individuals because of the complexity, duration, and range of medical, behavioral, environmental, and social needs. There is a growing evidence base showing that family-centered active management approaches that include activation and empowerment of care partners are well suited to improve care quality, health-related outcomes, and healthcare costs. Existing ADRD care management models have had limited translation to real world practice to date. This presentation will outline key experiences and lessons learned after a decade of work developing, adapting and embedding a comprehensive family-focused care management model called MIND at Home into practice. The work, supported in part by the NIA IMPACT Collaboratory, partners with Centene Corporation, a large managed care organization, and the American Medical Group Association, a non-profit trade organization representing multi-specialty groups and integrated health systems, to implement MIND at Home in different health care settings. MIND at Home implementation illustrates three overriding principles: (1) the necessity of “meeting people and health systems where they are” (literally and figuratively), (2) the importance of effectively matching intervention to outcome and context, and (3) contributions of key partner and stakeholders for adaptation and successful program evolution. Key considerations in implementation include engagement of staff, persons living with dementia, and care partners; program eligibility and referrals; ongoing staff training and support; organic drift over time; role clarity; and sustainability. Program flexibility and adaptation in light of individual, family, and system complexity is essential for successful implementation and sustainability.

